# Beyond PSA—Can Systemic Inflammatory Indices Improve Prostate Cancer Detection?

**DOI:** 10.3390/medsci14020243

**Published:** 2026-05-07

**Authors:** Marius Ivănuță, Abdallah Shurrab, Dragoș Puia, Ana-Maria Ivănuță, Mihaela Corlade-Andrei, Nicolae Stoican, Cătălin Pricop

**Affiliations:** 1 Grigore T. Popa University of Medicine and Pharmacy Iasi, 700115 Iasi, Romania; marius.ivanuta@umfiasi.ro (M.I.); abdo_l2001@hotmail.com (A.S.); catalin.pricop@umfiasi.ro (C.P.); 2 Department of Urology, “Dr. C.I. Parhon” Clinical Hospital, 700503 Iasi, Romania; 3 Emergency Care Department, “Sf. Spiridon” County University Emergency Hospital, 700111 Iasi, Romania; ion.ana-maria@d.umfiasi.ro; 4 Department of Urology, “Sf. Ioan cel Nou” County Emergency Hospital, 720033 Suceava, Romania; nicustoican@yahoo.com

**Keywords:** prostate cancer, prostate-specific antigen, inflammatory biomarkers, systemic inflammatory indices, neutrophil-to-lymphocyte ratio, systemic immune–inflammation index, aggregate index of systemic inflammation, prostate biopsy, diagnostic accuracy

## Abstract

Background: Prostate-specific antigen (PSA) remains the primary biomarker for prostate cancer detection; however, its limited specificity leads to unnecessary biopsies. Systemic inflammatory indices derived from routine blood tests have been proposed as potential adjunctive diagnostic tools. This study aimed to evaluate the diagnostic performance of these indices in patients undergoing prostate biopsy for suspected prostate cancer. Methods: A retrospective observational study was conducted, including 307 patients who underwent transrectal ultrasound-guided prostate biopsy for elevated PSA between January 2021 and January 2023. Patients were classified as benign prostatic hyperplasia (BPH) or prostate cancer (PCa) based on histopathological findings. Inflammatory indices, including neutrophil-to-lymphocyte ratio (NLR), systemic immune–inflammation index (SII), Systemic Inflammation Response Index (SIRI), and aggregate index of systemic inflammation (AISI), were calculated. Logistic regression and receiver operating characteristic (ROC) analyses were performed to assess diagnostic performance. Results: PCa was diagnosed in 65.1% of patients. Several inflammatory indices were significantly higher in the PCa group. Among them, AISI showed diagnostic performance comparable to PSA. The integration of inflammatory indices into multivariable models improved predictive accuracy, with the combined model demonstrating the highest discriminative ability. In contrast, these markers showed limited capacity in distinguishing tumour aggressiveness. Conclusions: Systemic inflammatory indices are associated with prostate cancer presence and may enhance diagnostic performance when used alongside PSA. However, their role remains complementary, as they provide limited value in risk stratification and should not be considered standalone diagnostic tools.

## 1. Introduction

Prostate cancer (PCa) represents a major global malignancy among men. Within Europe, it is the most frequently diagnosed cancer in the male population and constitutes the third leading cause of cancer-related mortality [1,2]. Significant advancements have been noted in early diagnostic methodologies during the previous decades. Prostate-specific antigen (PSA) has emerged as a low-cost, widely available and effective biomarker in diagnostic and screening purposes of PCa. Despite these wide applications of PSA, it has various limitations that limit its diagnostic accuracy. PSA harbours low specificity because it is also secreted in other prostate-related pathologies (BPH), prostatitis and UTIs [3,4]. Furthermore, other significant concerns such as grey-zone dilemma, overdiagnosis and overtreatment were also documented [5]. As a result, this clinical uncertainty usually leads to the decision for a confirmatory prostate biopsy, many of which may be considered as “unnecessary” and carry undesired complications for the patient. It was approximated that three out of four persons that have elevated PSA do not ultimately have PCa [6]. Hence, the need and pursuit to find novel tools with enhanced diagnostic accuracy is imperative.

Systemic inflammation has a pivotal role in cancer progression and the tumour’s microenvironment [7,8]. Thereupon, circulating inflammatory markers and calculated immune–inflammatory ratios offer a practical and economical approach for evaluating inflammatory status, as they are derived from routine, widely accessible complete blood count (CBC) parameters and other standard assays. Different inflammatory biomarkers (CRP, neutrophil–lymphocyte ratio (NLR), monocyte–lymphocyte ratio (MLR), platelet–lymphocyte ratio (PLR)) are identified as indicators of poor prognosis and pathological aggressiveness in various malignant tumours [9,10,11]. Furthermore, growing evidence points out the enhanced diagnostic ability of these immune–inflammatory indices in distinct cancers [12,13]. In PCa, the prognostic value of inflammatory–immune indices is already well-known [14]. However, their diagnostic value in men undergoing prostate biopsy due to elevated PSA remains unclear. The data in the literature are relatively sparse and heterogeneous, which opens the door to compelling questions about their possible utility in early diagnosis and screening pursuits. Moreover, most investigations focus on isolated markers and lack comprehensive, head-to-head comparisons of multiple indices within the same cohort. Standardised thresholds for clinical implementation are also absent. Collectively, these limitations underscore a critical gap in validating inflammatory indices as adjunctive diagnostic tools in the PSA-elevated biopsy population, as their definitive diagnostic contribution remains incompletely defined.

This present study aims to investigate the potential role of routinely measured blood-derived inflammatory hematologic ratios, specifically NLR, MLR, PLR, systemic immune–inflammation index (SII) and Systemic Inflammation Response Index (SIRI), as adjunctive diagnostic markers in men undergoing prostate biopsy with elevated PSA for suspected prostate cancer. The main objective is to determine whether these indices significantly offer diagnostic information beyond PSA by comparing their distributions between patients with malignant and benign biopsy findings. In addition, this study aims to explore the relationship between inflammatory ratios and pathological disease characteristics including Gleason score and ISUP grade group among individuals with histologically confirmed prostate cancer, to assess if systemic inflammatory status is associated with tumour aggressiveness at initial diagnosis.

## 2. Materials and Methods

### 2.1. Study Design and Patient Selection

We conducted a retrospective observational study at the Urology Clinic of “Dr. C. I. Parhon” Clinical Hospital, Iași, Romania. The study included consecutive patients who underwent transrectal ultrasound-guided prostate biopsy for elevated PSA levels and clinical suspicion of PCa, between January 2020 and January 2023.

Elevated PSA levels represented the primary indication for prostate biopsy in this cohort. Given the non-specific nature of PSA, histopathological examination was considered the reference standard for definitive diagnosis.

Patients were considered eligible if complete pre-biopsy laboratory data were available. To ensure consistency between laboratory evaluation and histopathological diagnosis, only patients who had a CBC performed within five days prior to prostate biopsy were included. Patients with laboratory tests obtained more than five days before biopsy were excluded to limit temporal variability in inflammatory markers and better reflect the systemic inflammatory status at the time of tissue sampling.

### 2.2. Data Collection and Pathological Assessment

Demographic data and laboratory parameters were retrieved from the hospital’s electronic medical records.

All biopsy specimens were evaluated by experienced genitourinary pathologists according to standard pathological criteria. Based on histopathological findings, patients were classified into two groups: BPH and PCa.

In patients diagnosed with PCa, tumour aggressiveness was assessed using the Gleason scoring system and categorised according to the International Society of Urological Pathology (ISUP) grading classification.

### 2.3. Exclusion Criteria

To minimise potential confounding related to systemic inflammatory conditions, patients with factors known to independently influence peripheral blood inflammatory parameters were excluded. Specifically, we excluded patients with:

Active urinary tract infection at the time of laboratory testing or biopsy.

Acute infectious or septic conditions.

Other active malignancies.

Chronic inflammatory or autoimmune diseases.

Ongoing systemic corticosteroid or immunosuppressive treatment.

Recent surgery or acute trauma prior to laboratory assessment.

Incomplete laboratory data required for the calculation of inflammatory indices.

CBC testing was performed outside the predefined five-day interval before biopsy.

### 2.4. Prostate Biopsy Procedure

All prostate biopsies were performed under transrectal ultrasound (TRUS) guidance, according to the institutional standard protocol. The same ultrasound system (BK Medical BK-2000, BK Medical, Herlev, Denmark) was used for all procedures to ensure methodological uniformity. The procedure was carried out with the patient in the lithotomy position. After appropriate antiseptic preparation, the transrectal ultrasound probe was introduced, and systematic prostate sampling was performed under real-time ultrasound guidance.

A standardised 12-core systematic biopsy scheme was applied to all patients.

All biopsy specimens were immediately fixed in formalin and processed within the hospital’s Histopathology Laboratory. Histopathological examination was conducted by experienced pathologists in accordance with established diagnostic criteria.

### 2.5. Inflammatory Indices

Complete blood count was performed using a Sysmex XN-1000 analyser (Sysmex Corporation, Kobe, Japan), while PSA levels were measured using the ARCHITECT i1000SR and Alinity analysers (Abbott Laboratories, Abbott Park, IL, USA). All analyses were conducted at “Dr. C.I. Parhon” Clinical Hospital.

The following CBC-derived inflammatory indices were calculated:

Neutrophil-to-Lymphocyte Ratio (NLR):

$$NLR=\frac{lymphocyte\;count}{neutrophil\;coun}$$

Monocyte-to-Lymphocyte Ratio (MLR):

$$MLR=\frac{monocyte\;count}{lymphocyte\;count}$$

Systemic Immune–Inflammation Index (SII):

$$PLR=\frac{platelet\;count}{lymphocyte\;count}$$

Systemic Inflammation Response Index (SIRI):

$$SII=platelet\;count\times \frac{neutrophil\;count}{lymphocyte\;count}$$

Aggregate Index of Systemic Inflammation (AISI):

$$SIRI=\frac{monocyte\;count\times neutrophil\;count}{lymphocyte\;count}$$

$$AISI=\;\frac{\;neutrophil\;count\times monocyte\;count\times platelet\;count}{lymphocyte\;count}$$

All haematological parameters were obtained from peripheral venous blood samples collected as part of routine pre-biopsy evaluation.

### 2.6. Ethical Approval

The study protocol was approved by the Ethics Committee of “Dr. C. I. Parhon” Clinical Hospital, Iași, Romania (Approval No. 9400/02.10.2025). The study was conducted in accordance with the principles of the Declaration of Helsinki.

### 2.7. Statistical Analysis

Statistical analysis was performed using SPSS software, version 26.0 (SPSS Inc., Chicago, IL, USA). Continuous variables were reported as mean ± standard deviation, while categorical variables were presented as counts and percentages. Group comparisons were conducted using the independent-samples *t*-test for continuous variables and the chi-square test for categorical variables. Variables with *p* < 0.05 in univariable analysis were considered for inclusion in multivariable models.

To evaluate the association between CBC-derived inflammatory indices and prostate cancer diagnosis, logistic regression models were used. The discriminative performance of the inflammatory markers was assessed using receiver operating characteristic (ROC) curve analysis, and the area under the curve (AUC) was calculated. A *p*-value < 0.05 was considered statistically significant. Cutoff values were determined using the Youden index derived from ROC curve analysis.

Model performance was assessed using both discrimination and overall accuracy measures. Discrimination was evaluated using the area under the receiver operating characteristic curve (AUROC). In addition, model performance was further quantified using the Brier score, which reflects the mean squared difference between predicted probabilities and observed outcomes, with lower values indicating better model performance.

The Brier score was calculated for each model based on predicted probabilities obtained from logistic regression analyses. Calibration was additionally assessed using smooth, nonparametric calibration plots.

## 3. Results

During the study period (January 2020–January 2023), a total of 703 TRUS prostate biopsies were performed in patients with elevated serum PSA levels and clinical suspicion of PCa at our institution.

**Figure 1:**
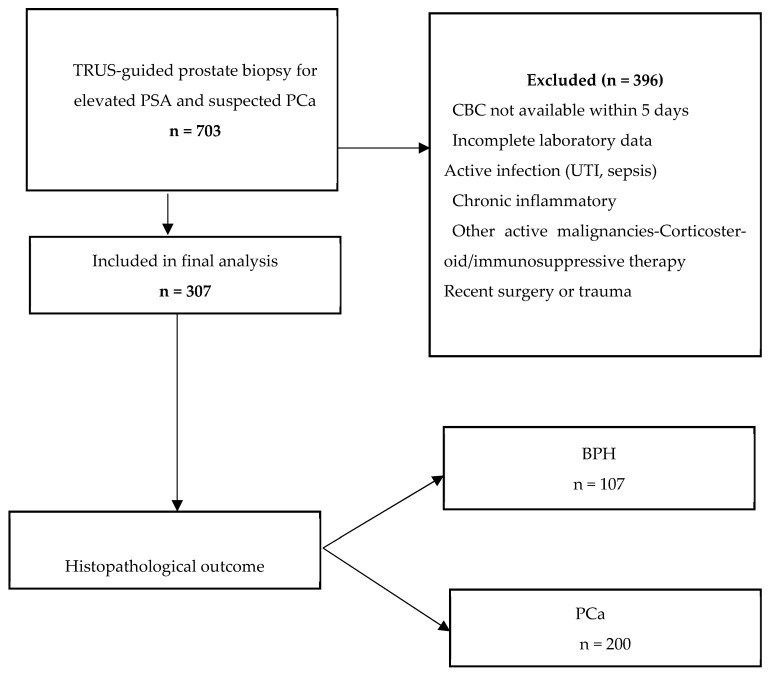
Flowchart of patient selection and study population.

After applying the predefined inclusion and exclusion criteria, 307 patients met the eligibility criteria and were included in the final statistical analysis. The patient selection process is illustrated in Figure 1.

The baseline characteristics of the study population are summarised in Table 1. Among the 307 included patients, 107 (34.9%) were diagnosed with BPH and 200 (65.1%) with prostate cancer.

The mean age of the overall cohort was 73.57 ± 8.52 years. Patients diagnosed with PCa were older than those with BPH (75.01 ± 7.97 vs. 70.89 ± 8.49 years), although the difference did not reach statistical significance (*p* = 0.06).

Serum PSA levels were significantly higher in the PCa group compared to the BPH group (47.25 ± 86.40 vs. 8.49 ± 4.21 ng/mL, *p* < 0.001).

Comparative analysis of laboratory parameters and inflammatory indices between the BPH and prostate cancer groups revealed significant differences in several haematological and inflammatory markers, as detailed in Table 2.

Univariable logistic regression analysis was performed to assess the association between clinical and laboratory parameters and the presence of prostate cancer. Several variables, including age, PSA, haemoglobin, and inflammatory indices, showed significant associations. The detailed results are presented in Table 3.

Multivariable logistic regression analysis was performed to identify independent predictors of prostate cancer, and we constructed three models. The baseline model included age and PSA, while Models 2 and 3 additionally incorporated AISI and NLR, respectively.

The inclusion of inflammatory indices improved model performance compared to the baseline model. The most pronounced improvement was observed when AISI was added, resulting in higher explanatory power and better overall model fit. In this model, all variables remained statistically significant. In contrast, although the model including NLR also showed improved performance, the effect was less pronounced, and age no longer retained statistical significance.

Overall, AISI provided the greatest incremental value over the baseline model, as shown in Table 4. In addition to discrimination and model fit, overall predictive accuracy was evaluated using the Brier score. The model incorporating age, PSA, and AISI demonstrated the best performance (Brier score = 0.112), followed by the model including NLR (Brier score = 0.131), while the baseline model showed lower performance (Brier score = 0.150).

**Figure 2:**
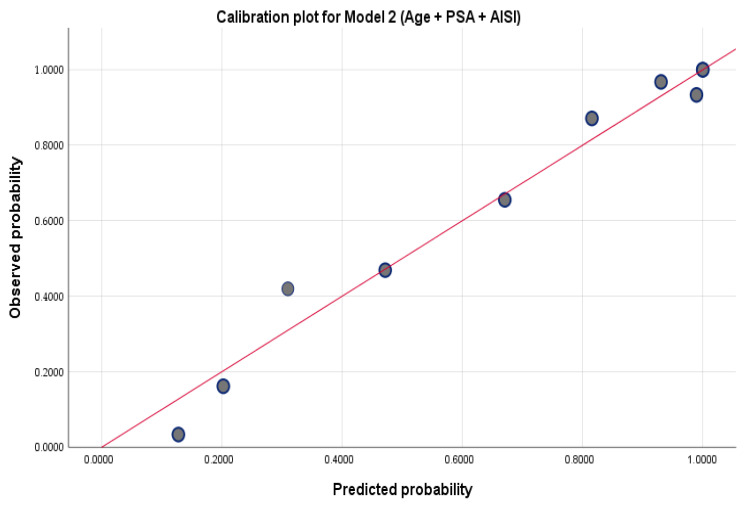
Calibration plot for Model 2. The dots represent observed probabilities, and the red line represents the ideal calibration.

In Figure 2, the calibration plot for Model 2 demonstrates good agreement between predicted and observed probabilities across deciles, indicating adequate model calibration.

**Figure 3:**
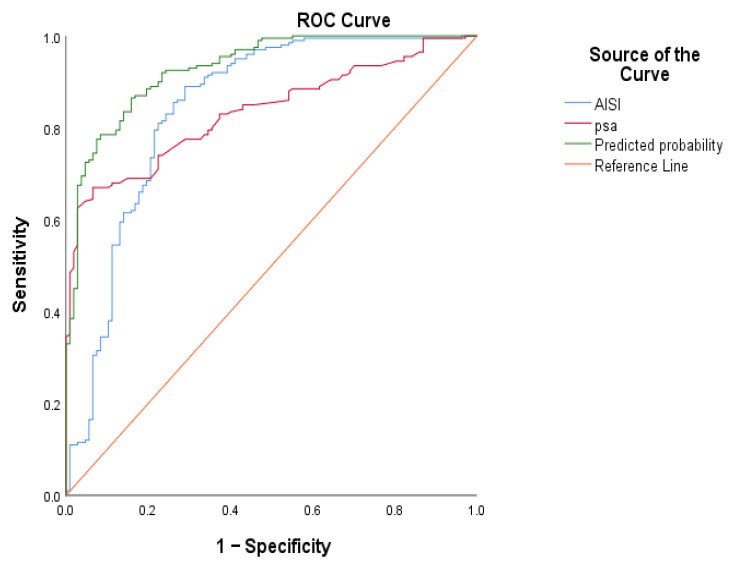
ROC curves for PSA, AISI, and the combined model (age + PSA + AISI) in predicting prostate cancer.

ROC curve analysis was used to assess the ability of PSA, AISI, and the combined model to discriminate between patients with and without prostate cancer, as shown in Figure 3. The combined model showed the best overall performance, with an AUC of 0.930. AISI showed a slightly higher AUC than PSA (0.842 vs. 0.835), suggesting comparable discriminative capacity. Notably, incorporating AISI into the baseline model was associated with a clear improvement in predictive performance.

Exploratory cutoff values for PSA and the investigated inflammatory indices were determined using ROC curve analysis based on the maximum Youden index. The corresponding sensitivity, specificity, and AUC values are presented in Table 5.

Stratification by tumour grade revealed consistent differences across both clinical and inflammatory parameters, as detailed in Table 6. Patients with high-grade disease exhibited higher PSA levels alongside a more pronounced inflammatory profile, reflected by increased SII, SIRI, and AISI values. A similar trend was observed for NLR, although this did not reach statistical significance.

**Figure 4:**
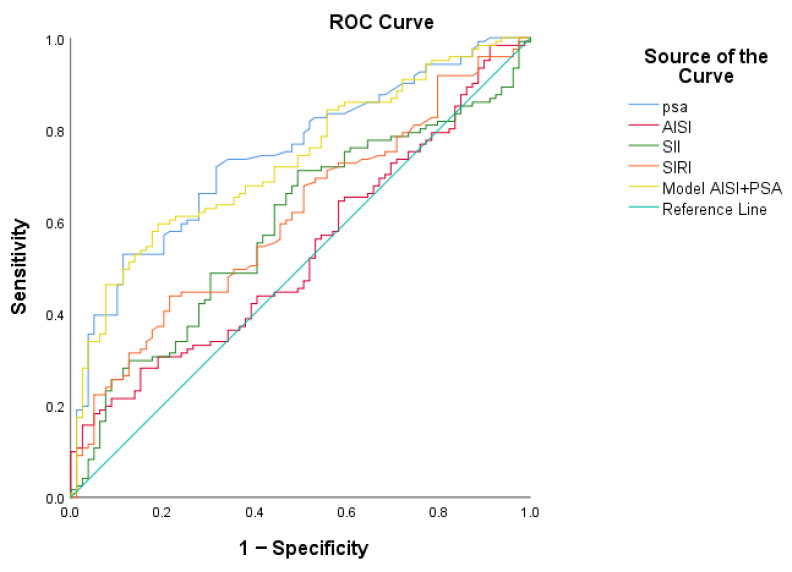
ROC curves illustrating the diagnostic performance of PSA, AISI, SII, SIRI, and the combined model in differentiating low-grade from high-grade prostate cancer.

The discriminatory ability of inflammatory indices for differentiating between low- and high-grade disease was further assessed using ROC curve analysis, as shown in Figure 4. PSA demonstrated the highest performance, with an AUC of 0.744. Inflammatory markers showed limited predictive value, with AISI exhibiting the weakest discriminatory capacity. The addition of AISI to PSA did not improve model performance, as reflected by a slightly lower AUC of 0.732.

## 4. Discussion

In our cohort, several inflammation-derived indices, particularly SII, SIRI, and AISI, were significantly elevated in patients with prostate cancer, indicating an association between systemic inflammatory activation and malignant disease. Importantly, these markers demonstrated a diagnostic performance comparable to PSA, with AISI showing similar discriminative capacity. Notably, AISI showed the most favourable balance between sensitivity and specificity among the inflammatory indices, whereas PLR and SII had limited discriminatory value. Therefore, these markers may be more useful as components of multivariable predictive models rather than as standalone diagnostic thresholds. Their most notable contribution emerged when incorporated into multivariable models, where they significantly improved predictive performance, yielding excellent overall discrimination. These findings suggest that inflammatory indices provide meaningful incremental diagnostic value rather than functioning as standalone biomarkers. The interpretation of PSA levels in this cohort requires careful consideration, as PSA is a non-specific biomarker that may be elevated in a range of benign and inflammatory conditions, including BPH, prostatitis, or urinary tract infections. This is particularly relevant in a biopsy-selected population, where PSA elevation serves as the primary indication for further investigation. Consequently, the elevated and highly variable PSA values observed in our study likely reflect the underlying clinical heterogeneity rather than malignancy alone. However, their performance was less consistent in risk stratification, as reflected in their limited ability to discriminate tumour aggressiveness. In this context, these markers likely reflect tumour-associated systemic inflammatory response rather than serving as reliable tools for grading disease severity.

Our findings reflect the complex interplay between tumour biology and systemic inflammation. Chronic inflammation is increasingly recognised as a key contributor to prostate carcinogenesis, promoting genomic instability, cellular proliferation, and tumour progression through multiple molecular pathways [14,15,16].

Although inflammation-based biomarkers are primarily described in the literature as prognostic indicators, our results suggest that they may also provide diagnostic value, particularly when integrated into multivariable predictive models [17]. Similarly, other inflammatory markers have demonstrated limited ability to differentiate between benign and malignant conditions or between different risk categories of prostate cancer [18].

In the present cohort, although SII, SIRI, and AISI were significantly higher in high-grade tumours, their ROC curve analysis revealed limited discriminatory capacity. Notably, AISI showed minimal predictive value, and its addition to PSA did not improve model performance. This suggests that composite inflammatory indices, despite integrating multiple haematological parameters, may not provide additional clinical benefit in distinguishing tumour aggressiveness beyond what is already achieved by PSA.

Our observations are consistent with broader oncological evidence indicating that inflammatory markers are best interpreted as complementary biomarkers. In other urological malignancies, such as testicular cancer, inflammatory indices have been shown to enhance diagnostic and prognostic assessment when used alongside classical tumour markers, particularly in equivocal cases [19,20].

The results regarding NLR strongly corroborate existing literature. Previous studies demonstrated that an elevated pre-biopsy NLR is an independent risk factor for PCa and effectively predicts positive biopsy outcomes. Building on the utility of simple cellular ratios, our data also validates the more comprehensive multi-cell indices. Moreover, a retrospective cohort emphasised the value of NLR and SII as independent risk factors for PCa [21,22]. Similarly, our observation highlighting that AISI serves as a significant univariable predictor is highly consistent with recent research by Liu et al., which established that blood inflammatory markers, particularly AISI, offer enhanced diagnostic accuracy for differentiating BPH from PCa. These composite markers theoretically provide a more robust reflection of the immune–inflammatory cascade by incorporating monocytes and platelets, which act to facilitate tumour cell survival and protect circulating malignant cells from immune surveillance [10]. 

Conversely, our analysis revealed no significant difference in MLR between the PCa and BPH cohorts, a finding that diverges from several prior investigations. Studies such as those by Eren have previously identified MLR as a significant independent predictor of PCa [23,24]. This discrepancy may stem from variations in cohort demographics and differences in the prevalence of concurrent asymptomatic inflammatory conditions. Additionally, our rigorous exclusion criteria, which narrowed the initial 703 biopsies to 307 to ensure precise CBC availability and timing, may have reduced the statistical power needed to detect subtle diagnostic differences in monocyte-driven ratios.

From a clinical standpoint, the primary advantage of utilising markers like NLR, SIRI, and AISI lies in their high accessibility and cost-effectiveness. Because these indices are derived from routine pre-biopsy blood tests, they incur no additional financial burden on healthcare systems. Integrating these parameters into modern risk-stratification models has the potential to substantially reduce the incidence of negative biopsies, thereby sparing patients from procedure-associated anxiety, discomfort, and the risk of urosepsis.

Several limitations of the present study should be acknowledged. The retrospective design may introduce selection bias and limits the ability to establish causal relationships. In addition, MRI-guided prostate biopsy was not available at our institution during the study period, and all patients underwent systematic TRUS biopsy. This may have influenced both tumour detection and grading accuracy. Given that MRI-targeted biopsy improves the detection of clinically significant prostate cancer while reducing the identification of indolent lesions [25,26], the use of systematic biopsy alone may have affected patient classification in our cohort.

This limitation is relevant when interpreting the diagnostic performance of inflammatory indices, as some degree of misclassification cannot be excluded. Consequently, the observed associations between these markers and prostate cancer should be interpreted with caution. Future studies incorporating MRI-targeted biopsy are needed to provide more accurate correlations between inflammatory markers and clinically significant disease.

MRI-targeted biopsy has since been implemented in routine clinical practice at our institution, potentially enabling more accurate evaluation of these markers in future analyses.

Furthermore, systemic inflammatory indices may be influenced by a variety of confounding factors, including subclinical infections, comorbidities, or individual immune variability, which were not fully controlled in this analysis.

## 5. Conclusions

Our study supports the association between systemic inflammatory indices and the presence of prostate cancer, highlighting their potential role within the diagnostic framework of patients with elevated PSA. While these markers do not outperform established tools when used in isolation, their integration into combined models may enhance diagnostic accuracy. However, their limited ability to discriminate tumour aggressiveness restricts their applicability in risk stratification. In this context, inflammatory indices should be regarded as adjunctive biomarkers that reflect tumour-associated systemic response rather than as independent determinants of disease severity.

## Figures and Tables

**Table 1:** Baseline clinical characteristics of the study population.

Variable	Total (*n* = 307)	BPH (*n* = 107)	PCa (*n* = 200)	*p*-Value
Mean Age (SD)	73.57 (8.52)	70.89 (8.49)	75.01 (7.97)	0.06
PSA (SD)	33.71 (72.1)	8.49 (4.21)	47.25 (86.4)	0.001

**Table 2:** Comparison of laboratory parameters and inflammatory indices between BPH and prostate cancer.

Variable	Total (*n* = 307)	BPH (*n* = 107)	PCa (*n* = 200)	*p*-Value
General Laboratory Parameters				
Haemoglobin (g/dL), mean ± SD		14.75 (±1.46)	13.70 (±2.322)	0.001
Creatinine (mg/dL), mean ± SD		0.99 (±0.35)	1.26 (±2.32)	0.248
Urea (mg/dL), mean ± SD		38.29 (±14.59)	45.44 (±30.03)	0.005
Complete Blood Count Parameters				
Neutrophils (×10^3^/µL), mean ± SD		4238.63 (±1456.94)	5048.85 (±119.37)	0.001
Lymphocytes (×10^3^/µL), mean ± SD		1840.09 (±573.10)	1900.45 (±675.11)	0.25
Monocytes (×10^3^/µL), mean ± SD		620.19 (±257.6)	650.1 (±228.25)	0.297
Platelets (×10^3^/µL), mean ± SD		224.2 (±58.21)	231.9 (±70.51)	0.309
Inflammatory Indices				
NLR, mean ± SD		2.56 (±1.41)	4.87 (±3.63)	0.001
MLR, mean ± SD		0.36 (±0.17)	0.37 (±0.17)	0.354
SIRI, mean ± SD		1.7 (±1.9)	2.4 (±1.9)	0.006
AISI, mean ± SD		260.2 (±197.57)	502.67 (±219.02)	0.001
PLR, mean ± SD		119.4 (±45)	127 (±70)	0.25

**Table 3:** Univariable logistic regression analysis for prediction of prostate cancer.

Variable	B	OR (95% CI)	*p*-Value
Age	0.063	1.065 (1.033–1.099)	<0.001
PSA	0.180	1.198 (1.125–1.275)	<0.001
Haemoglobin	−0.280	0.756 (0.658–0.867)	<0.001
Platelets	0.002	1.002 (0.998–1.005)	0.336
Neutrophils	0.000	1.000 (1.000–1.001)	<0.001
Lymphocytes	0.000	1.000 (1.000–1.001)	0.432
Monocyte	0.001	1.000 (1.000–1.001)	0.297
Creatinine	0.343	1.409 (0.775–2.563)	0.261
NLR	0.478	1.612 (1.346–1.931)	<0.001
PLR	0.005	1.005 (1.000–1.009)	0.045
SII	0.002	1.002 (1.001–1.002)	<0.001
SIRI	0.307	1.359 (1.091–1.694)	0.006
AISI	0.007	1.007 (1.005–1.009)	<0.001

**Table 4:** Multivariable logistic regression models for prediction of prostate cancer.

Variable	Model 1 (Age + PSA) OR (95% CI)	*p*	Model 2 (Age + PSA + AISI) OR (95% CI)	*p*	Model 3 (Age + PSA + NLR) OR (95% CI)	*p*
Age	1.033 (0.996–1.073)	0.085	1.055 (1.008–1.104)	0.022	1.024 (0.985–1.066)	0.235
PSA	1.187 (1.116–1.263)	<0.001	1.168 (1.092–1.248)	<0.001	1.174 (1.101–1.253)	<0.001
AISI	—	—	1.006 (1.004–1.008)	<0.001	—	—
NLR	—	—	—	—	1.461 (1.215–1.757)	<0.001
Model performance	Model 1		Model 2		Model 3	
−2 Log Likelihood	270.4		205.9		242.9	
Nagelkerke R^2^	0.466		0.638		0.544	
Brier Score	0.150		0.112		0.131	

Lower Brier scores indicate better model performance.

**Table 5:** Diagnostic performance of PSA and inflammatory indices based on ROC curve analysis.

Variable	Cut-Off	Sensitivity (%)	Specificity (%)
PSA	12.85	67.0	93.5
AISI	279.01	84.5	71.0
NLR	3.32	53.5	84.1
SIRI	1.34	73.0	55.1
SII	760.39	40.0	88.8
PLR	165.59	21.0	91.6

**Table 6:** Comparison of clinical and inflammatory parameters between low-grade and high-grade prostate cancer.

Variable	Low-Grade (n = 79)	High-Grade (n = 121)	*p*-Value
Age (years), mean ± SD	73.67 ± 6.10	75.88 ± 8.90	0.038
PSA (ng/mL), mean ± SD	27.50 ± 78.59	60.15 ± 89.20	0.007
NLR, mean ± SD	4.28 ± 3.75	5.26 ± 3.51	0.066
SII, mean ± SD	643.88 ± 397.45	785.39 ± 498.51	0.027
SIRI, mean ± SD	2.01 ± 1.82	2.58 ± 1.97	0.035
AISI, mean ± SD	467.63 ± 197.43	538.24 ± 301.56	0.047

## Data Availability

Data is contained within the article.

## Abbreviations

The following abbreviations are used in this manuscript:

PCa	Prostate Cancer
PSA	Prostate-Specific Antigen
BPH	Benign Prostatic Hyperplasia
UTI	Urinary Tract Infection
CBC	Complete Blood Count
NLR	Neutrophil-to-Lymphocyte Ratio
MLR	Monocyte-to-Lymphocyte Ratio
PLR	Platelet-to-Lymphocyte Ratio
SII	Systemic Immune–Inflammation Index
SIRI	Systemic Inflammation Response Index
AISI	Aggregate Index of Systemic Inflammation
TRUS	Transrectal Ultrasound
ISUP	International Society of Urological Pathology
ROC	Receiver Operating Characteristic
AUC	Area Under the Curve

## References

[1] Elmadani M., Mokaya P.O., Omer A.A.A., Kiptulon E.K., Klara S., Orsolya M. (2025). Cancer Burden in Europe: A Systematic Analysis of the GLOBOCAN Database (2022). BMC Cancer.

[2] Cornford P., van den Bergh R.C.N., Briers E., Van den Broeck T., Brunckhorst O., Darraugh J., Eberli D., De Meerleer G., De Santis M., Farolfi A. (2024). EAU-EANM-ESTRO-ESUR-ISUP-SIOG Guidelines on Prostate Cancer—2024 Update. Part I: Screening, Diagnosis, and Local Treatment with Curative Intent. Eur. Urol..

[3] Sekhoacha M., Riet K., Motloung P., Gumenku L., Adegoke A., Mashele S. (2022). Prostate Cancer Review: Genetics, Diagnosis, Treatment Options, and Alternative Approaches. Molecules.

[4] Khan T., Altamimi M.A., Hussain A., Ramzan M., Ashique S., Alhuzani M.R., Alnemer O.A., Khuroo T., Al-shammari H.A. (2022). Understanding of PSA Biology, Factors Affecting PSA Detection, Challenges, Various Biomarkers, Methods, and Future Perspective of Prostate Cancer Detection and Diagnosis. Adv. Cancer Biol. Metastasis.

[5] Oh C., Kang H. (2025). The Effectiveness and Harms of PSA-Based Prostate Cancer Screening: A Systematic Review. Healthcare.

[6] Cancer Research UK. https://www.cancerresearchuk.org/health-professional/diagnosis/investigations/psa-testing.

[7] Heshmat-Ghahdarijani K., Sarmadi V., Heidari A., Falahati Marvasti A., Neshat S., Raeisi S. (2023). The Neutrophil-to-Lymphocyte Ratio as a New Prognostic Factor in Cancers: A Narrative Review. Front. Oncol..

[8] Zhang L., Fu J., Liu X., Feng S., Leng Y. (2025). The Immune Landscape of Systemic Inflammation in Prostate Cancer. Cancer Biol. Med..

[9] Ma L., Yang F., Guo W., Tang S., Ling Y. (2024). Prognostic Role of Platelet-to-Lymphocyte Ratio in Patients with Rectal Cancer Undergoing Resection: A Systematic Review and Meta-Analysis. Front. Oncol..

[10] Liu X., Yin L., Shen S., Hou Y. (2023). Inflammation and Cancer: Paradoxical Roles in Tumorigenesis and Implications in Immunotherapies. Genes. Dis..

[11] Ozturk A.E., Komurcuoglu B., Karakurt G.K., Ozturk O. (2024). Prognostic Value of Diffuse Cancer Inflammation Index (ALI), Serum Neutrophil/Lymphocyte (NLR) and Platelet/Lymphocyte (PLR) in Advanced-Stage Lung Cancer. J. Cancer Res. Ther..

[12] Zheng J., Zheng L., Wang X., Mao X., Wang Q., Yang Y., Mo D. (2025). The Clinical Value of the Combined Detection of Systemic Immune-Inflammation Index (SII), Systemic Inflammation Response Index (SIRI), and Prognostic Nutritional Index (PNI) in Early Diagnosis of Gastric Cancer. J. Inflamm. Res..

[13] Islam M.M., Satici M.O., Eroglu S.E. (2024). Unraveling the Clinical Significance and Prognostic Value of the Neutrophil-to-Lymphocyte Ratio, Platelet-to-Lymphocyte Ratio, Systemic Immune-Inflammation Index, Systemic Inflammation Response Index, and Delta Neutrophil Index. Turk. J. Emerg. Med..

[14] Peng H., Luo X. (2019). Prognostic Significance of Elevated Pretreatment Systemic Inflammatory Markers for Patients with Prostate Cancer: A Meta-Analysis. Cancer Cell Int..

[15] Puia D., Ivănuță M., Pricop C. (2025). DNA methylation in bladder cancer: Diagnostic and therapeutic perspectives—A narrative review. Int. J. Mol. Sci..

[16] Oseni S.O., Naar C., Pavlović M., Asghar W., Hartmann J.X., Fields G.B., Esiobu N., Kumi-Diaka J. (2023). The molecular basis and clinical consequences of chronic inflammation in prostatic diseases: Prostatitis, benign prostatic hyperplasia, and prostate cancer. Cancers.

[17] Lorente D., Mateo J., Templeton A.J., Zafeiriou Z., Bianchini D., Ferraldeschi R., Bahl A., Shen L., Su Z., Sartor O. (2015). Baseline neutrophil–lymphocyte ratio (NLR) is associated with survival and response to treatment with second-line chemotherapy for advanced prostate cancer independent of baseline steroid use. Ann. Oncol..

[18] Puia D., Ivănuță M., Pricop C. (2025). Kidney Injury Molecule-1 as a Biomarker for Renal Cancer: Current Insights and Future Perspectives-A Narrative Review. Int. J. Mol. Sci..

[19] Kaynar M., Yildirim M.E., Gul M., Kilic O., Ceylan K., Goktas S. (2015). Benign prostatic hyperplasia and prostate cancer differentiation via platelet-to-lymphocyte ratio. Cancer Biomark..

[20] Puia D., Ivănuță M., Cauni V.M., Corlade-Andrei M., Pricop C. (2025). Complete Blood Count-Derived Inflammatory Markers and C-Reactive Protein in Testicular Cancer: Diagnostic and Prognostic Utility. Med. Sci..

[21] Kawahara T., Fukui S., Sakamaki K., Ito Y., Ito H., Kobayashi N., Izumi K., Yokomizo Y., Miyoshi Y., Makiyama K. (2015). Neutrophil-to-Lymphocyte Ratio Predicts Prostatic Carcinoma in Men Undergoing Needle Biopsy. Oncotarget.

[22] Eren H. (2020). Predictive Value of Neutrophil-to-Lymphocyte and Platelet-to-Lymphocyte Ratio Measured Prior to Prostate Biopsy. J. Urol. Surg..

[23] Wang S., Ji Y., Chen Y., Du P., Cao Y., Yang X., Ma J., Yu Z., Yang Y. (2022). The Values of Systemic Immune-Inflammation Index and Neutrophil–Lymphocyte Ratio in the Localized Prostate Cancer and Benign Prostate Hyperplasia: A Retrospective Clinical Study. Front. Oncol..

[24] Demirkol M.K., Barut O., Bilecan E.B., Şahinkanat T., Boran Ö.F., Metin M., Resim S. (2021). An Inflammatory Marker for Predicting Prostate Cancer in Prostate Biopsy: Monocyte-to-Lymphocyte Ratio. Yeni Urol. Derg..

[25] Schoots I.G., Ahmed H.U., Albers P., Asbach P., van den Bergh R.C., Godtman R.A., van Leeuwen P.J., Nordström T., Punwani S., Wallström J. (2025). Magnetic resonance imaging-based biopsy strategies in prostate cancer screening: A systematic review. Eur. Urol..

[26] Bratt O. (2026). Efficacy endpoints of MRI-based prostate cancer screening: An epidemiological perspective. Eur. Radiol..

